# Mechanical chest compression with LUCAS device does not improve clinical outcome in out-of-hospital cardiac arrest patients: A systematic review and meta-analysis

**DOI:** 10.1097/MD.0000000000017550

**Published:** 2019-11-01

**Authors:** Mao Liu, Zhuang Shuai, Jiao Ai, Kai Tang, Hui Liu, Jiankang Zheng, Junqi Gou, Zhan Lv

**Affiliations:** a Department of Cardiology, Cardiovascular Research Center, Affiliated Hospital of North Sichuan Medical College; b Department of Clinical Medicine, North Sichuan Medical College, Nanchong, Sichuan Province, P.R. China.

**Keywords:** cardiac arrest, cardiopulmonary resuscitation, LUCAS

## Abstract

**Background:**

Cardiac arrest (CA) is a serious threat to human health. Cardiopulmonary resuscitation (CPR) is an effective treatment for CA. Early and high-quality CPR is closely related to the survival rate of patients with CA. But manual chest compression has a lot of defects. To solve the defects and improve the quality of CPR, mechanical CPR device was invented. However, it has still controversy whether manual chest compression or mechanical chest compression is better. This systematic review was aimed to investigate the difference in clinical outcomes between manual chest compression and Lund University Cardiac Assist System (LUCAS) assisted CPR in patients with out-hospital CA.

**Methods:**

Original research studies, conducted on adult out-of-hospital CA, were included. PubMed/Medline, EMBASE, Scopus, Cochrane Library, CNKI, and Wanfang database were searched from the setting to February 21, 2019. Odds ratio (OR) with 95% confidence interval (CI) was selected as effect scale index for evaluation of the difference in return of spontaneous circulation (ROSC), survival to hospital admission, survival to hospital discharge, and survival to 30 days. Random effects model was used in this study to estimate overall mean effects.

**Results:**

A total of 6 articles, including 4 randomized controlled trials and 2 nonrandomized controlled trials, were selected. And 8501 subjects were involved to analyze the clinical outcomes of LUCAS and manual chest compression for patients with out-hospital CA. Comparisons of ROSC (33.3% vs 33.0%, *P* = .98; OR = 1; 95% CI: [0.89,1.13]), survival to hospital admission (22.7% vs 24.3%, *P* = .32; OR = 0.86; 95% CI: [0.65,1.15]), survival to hospital discharge (8.6% vs 10.7%, *P* = .50; OR = 0.92; 95% CI: [0.73,1.17]), and survival to 30 days (7.5% vs 8.5%, *P* = .50; OR = 0.92; 95% CI: [0.73,1.17]) were made. No significant difference was found.

**Conclusion:**

The synthesis of available evidence does not support that mechanical chest compression with LUCAS device improves clinical outcome in out-of-hospital CA patients compared with manual chest compression. Large scale studies with improved designs are still needed in the future.

## Abstract 2

Supplemental Digital Content is available in the text

## 1 Introduction

Cardiac arrest (CA) is the sudden suspension of cardiac ejection function. Previous retrospective studies have shown that in-hospital mortality of CA patients is 67% in adult and 5% in children.^[1]^ In the United States and Europe, about 300,000 and 450,000 people, respectively, suffer CA each year.^[2,3]^ Therefore, CA is a serious threat to human health. Cardiopulmonary resuscitation (CPR) is an effective treatment by using manual respiration to deliver air into the lung cavity, and simulating the heart pumping function through extra chest compression to supply oxygen to organs. Early and high-quality CPR is closely related to the survival rate of patients with CA.^[4,5]^ If CPR is done immediately after CA, the survival rate of patients is 63.6%. If the CPR is done in 5 minutes, the survival rate is 37.5%. However, if the time of CPR is more than 10 minutes, the survival rate is only 4.5%.^[6]^ The American Heart Association released a new CPR guideline which emphasizes high-quality CPR as the key to improve prognosis of CA patients.^[7,8]^ However, manual chest compression is limited by many factors such as the environment, the mental and physical strength of the rescuer. Some studies have shown that the quality of chest compression is difficult to guarantee during the movement of ambulances or stretchers.^[9,10]^ And it will be affected by fatigue of the rescuer.^[11]^

To solve the problems above and improve the quality of CPR, mechanical CPR device has been invented. According to the different pressing methods and ages, it can be divided into 3 types: point compression, load distribution (vest type) compression, and full chest (3D type) compression.^[12]^ In recent years, researchers have developed a variety of CPR devices, such as Thumper, AutoPulse, and the LUCAS chest compressors. These mechanical can provide patients with high-quality CPR through stable and long-lasting chest compression. In particular, the quality of chest compression during transportation has an outstanding advantages.^[13]^

LUCAS is a portable chest compression device designed to eliminate the problems of manual chest compression. Compared with manual chest compression, it has the following advantages: First, LUCAS saves manpower. LUCAS provides basic CPR, enabling rescuers to concentrate on advanced life support, improving efficiency, and speed of rescue, and thus improving patient survival rate. Second, LUCAS guarantees effectiveness. LUCAS keep the stabilization by setting parameters, such as frequency, depth, and rhythm, avoiding the deviation caused by manual chest compression. Therefore, it can guarantee the quality of CPR. Third, LUCAS removes fatigue factors and ensures the effectiveness of CPR. As mentioned above, manual chest compression for a long time can lead to the rescuers fatigue. It is difficult to guarantee the quality of CPR. The replacement of the operator in the middle will lead to the interruption of chest compression. However, LUCAS can avoid these problems and guarantee continuous life support.^[14]^ Nevertheless, some studies suggest that LUCAS is better than manual chest compression,^[15–17]^ but some studies do not support this opinion.^[18–21]^ The results of previous studies are inconsistent. Therefore, this study aims to comprehensively evaluate whether LUCAS can bring better clinical outcome than manual chest compression through meta-analysis.

## 2 Materials and methods

### 2.1 Search strategy

First, the main databases, including PubMed/Medline, EMBASE, Scopus, Cochrane Library, CNKI, and Wanfang database, were searched by computer. And then the references of related papers were searched twice to reduce the omission. The following search terms were used: mechanical, manual, chest compression, CA, cardiopulmonary resuscitation, and LUCAS. The publication time of the paper was set from the establishment of the database to February 21, 2019. The language is limited to English and Chinese.

### 2.2 Inclusion criteria

The original study types in this study included randomized controlled studies, cohort studies, or case-control studies. Subjects must include the LUCAS group and the manual chest compression group. The original paper must compare the CPR effects of the 2 groups of CA patients. If subgroup data is needed, the group with more cases is selected.

### 2.3 Exclusion criteria

In the following case, the articles will be excluded: animal studies, case reports, conference abstracts, reviews, drug trials, and languages other than Chinese or English, documents that do not have full text or incomplete data.

### 2.4 Review process

Two researchers independently screened and read the paper and extracted the data. The quality assessment was based on the Review Manager 5.3 bias score map. When the data extraction and quality evaluation encounter inconsistencies, the third researcher is invited to participate in the discussion after the discussion fails to reach an agreement.

### 2.5 Statistical analysis

The heterogeneity of each study was evaluated using the Cochrane *Q* test and *I*^2^ test. When *I*^2^ ≥ 50%, there was heterogeneity between studies. To minimize bias, all meta-analytical steps in this study were selected as random-effects model. The odds ratio (OR) and its 95% confidence interval (CI) is selected as the effect scale indicators. Funnel plot will be drawn to evaluate publication bias. At the same time, risk of bias assessment will be made according to Cochrane Collaboration's tool. A meta-regression will also be done to analyze the potential causes of bias. Except for the Cochrane *Q* test that the difference is statistically significant if *P* < .1. The others were defined as difference that is statistically significant if *P* < .05. Statistical analysis was performed using RevMan 5.3 software provided by the Cochrane Collaboration.

### 2.6 Ethical statement

This study was carried out in accordance with the recommendations in the preferred reporting items for systematic reviews and meta-analyses guidelines. Hence, the ethics committee or institutional review board permission is not needed.

## 3 Results

### 3.1 Basic information of included studies

**Figure 1:**
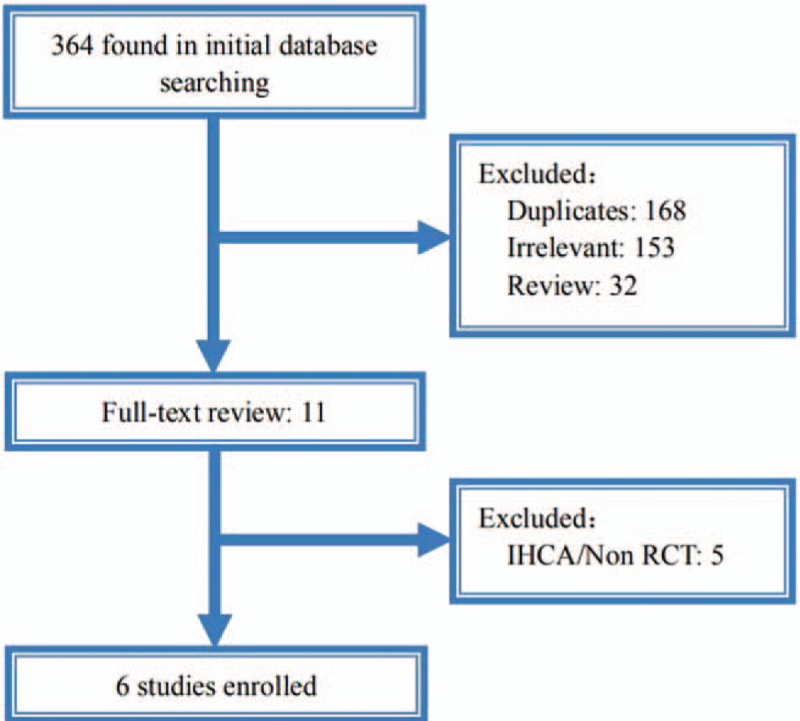
Screening process of the paper.

A total of 364 original study were obtained by searching the Chinese and English database. Three hundred fifty-three were excluded because of duplicate, irrelevant, and review after reading the texts and abstracts, there were 11 paper that basically satisfied the research topic. Further, 5 paper of them were excluded due to the research object were in-hospital patients with CA. Finally, 6 papers,^[18–23]^ including 4 randomized controlled trials and 2 nonrandomized cohort studies, were included. Baseline data are shown in Table 1. The paper search process is shown in Figure 1.

**Table 1:**
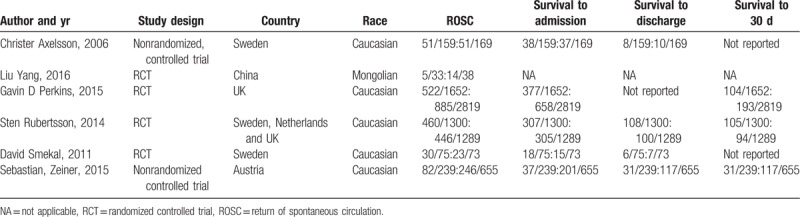
Main characteristic of included studies.

### 3.2 Return of spontaneous circulation

**Figure 2:**
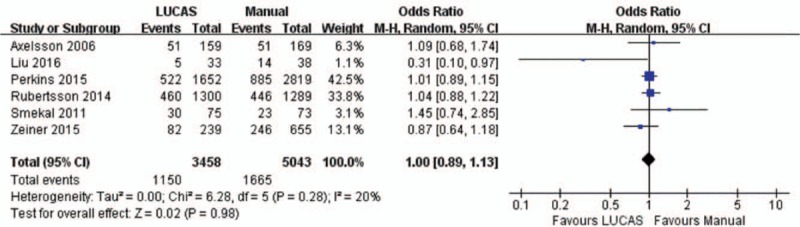
Forest plot of ROSC between LUCAS group and Manual group. ROSC = return of spontaneous circulation.

As shown in Figure 2, 6 studies with a total of 8501 subjects, compared the success rates of return of spontaneous circulation (ROSC) between the LUCAS group and the Manual group. The success rate of ROSC in the LUCAS group and the Manual group was similar, and the difference was not statistically significant (33.3% vs 33.0%, *P* = .98, OR = 1; 95% CI: [0.89, 1.13]).

### 3.3 Survival to hospital admission

**Figure 3:**
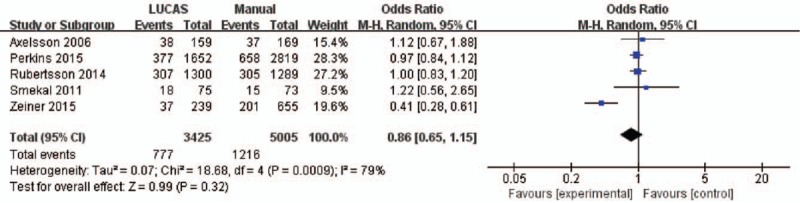
Forest plot of survival to hospital admission between LUCAS group and Manual group.

As shown in Figure 3, 8430 cases were enrolled, including 3425 cases in the LUCAS group and 5005 cases in the Manual group. There was no significant difference in survival to hospital admission between the 2 groups (22.7% vs 24.3%, *P* = .32; OR = 0.86; 95% CI: [0.65, 1.15]).

### 3.4 Survival to hospital discharge

**Figure 4:**
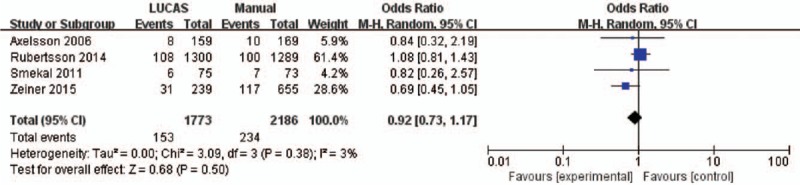
Forest plot of survival to hospital discharge between LUCAS group and Manual group.

As shown in Figure 4, 3959 cases were enrolled in 4 papers, including 1773 cases in the LUCAS group and 2186 cases in the Manual group. There was no significant difference in survival to hospital discharge between the 2 groups (8.6% vs 10.7%, *P* = .50; OR = 0.92; 95% CI: [0.73, 1.17]).

### 3.5 Survival to 30 days

**Figure 5:**
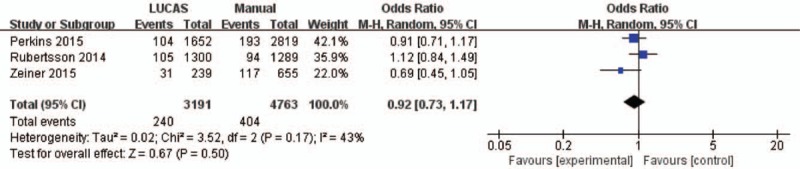
Forest plot of survival to 30 d between LUCAS group and Manual group.

As shown in Figure 5, 7954 cases were enrolled in 3 papers, including 2952 cases in LUCAS group and 4108 cases in Manual group. There was no significant difference between the 2 groups (7.5% vs 8.5%, *P* = .50; OR = 0.92; 95%CI: [0.73, 1.17]).

### 3.6 Quality evaluation and publication bias

**Figure 6:**
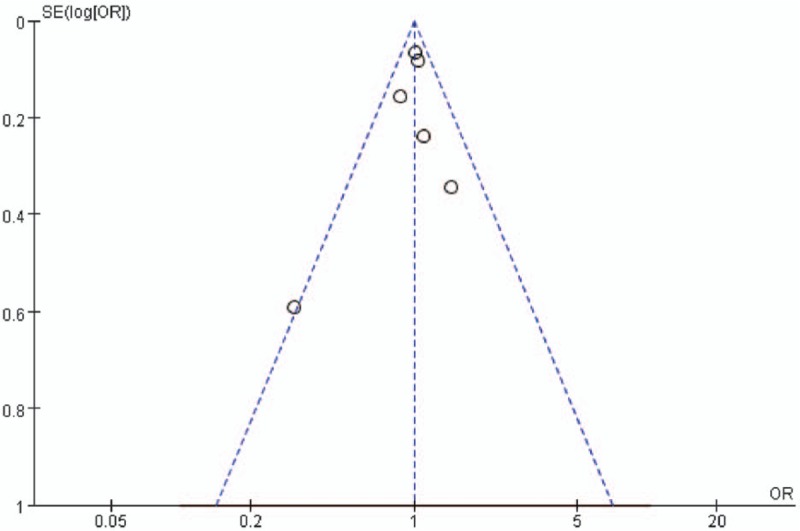
Funnel plot of the included paper.

In this study, each paper was scored using the Review Manager 5.3 bias analysis tool. Because of the need to rescue patients, double-blind design was impossible. As shown in Supplemental Digital Content (Suppl 1), each study had a low blind score, but had high scores in other indicators, and the sample size of the study was large, so this meta-analysis had relatively high quality. Moreover, the results of univariable meta-regression analysis were shown in Supplemental Digital Content (Suppl 2). The differences for sample size (*P* = .612), research type (*P* = .756), publication year (*P*= .774), and race (*P* = .536) did not significantly explain the heterogeneity. Also, the results of the funnel plot were shown in Figure 6. The included papers are distributed on both sides of the mid-line and the figure is relatively symmetrical. It are located at the tip of the funnel, indicating that this systematic review had high quality and small publication bias.

## 4 Discussion

### 4.1 Summary of study findings

This meta-analysis of 4 randomized controlled trials and 2 nonrandomized controlled trials evaluated the success rate and prognosis between LUCAS and manual CPR. It found that there was no meaningful difference between the experimental group and control group in ROSC, survival to hospital admission, survival to hospital discharge and survival to 30 days.

Some studies have shown that there is a direct relationship between the quality of chest compression and short-term survival. The American Heart Association guidelines and emergency cardiovascular care emphasize the high quality of CPR.^[24]^ However, manual chest compression will be affected by fatigue, especially after 2 to 3 minutes of CPR.^[11,25]^ It is difficult to maintain high-quality CPR. The replacement personnel will lead to CPR interruption, which will lead to a decline in CPR quality and affect prognosis of patients with CA.^[26–28]^ The use of mechanical chest compression can avoid these problems, thereby maintaining high-quality CPR, and even international guidelines published in 2010 that these devices can be seen as part of an overall strategy to improve the quality of CPR.^[24]^

LUCAS is a chest compression device that provides automatic chest compression and decompression CPR according to the principle of pneumatic. By improving the point pressing and the pressing head and adopting the suction disc press head, the chest can be pulled up while pressing it at the same time. It makes the thorax to fully rebound, causing a large negative pressure in the chest to promote blood return. Previous studies have shown that there is a controversy that whether LUCAS will bring better benefits to patients with CA. Existing experimental studies about LUCAS are controversial, but the results of relevant meta-analysis are generally consistent. A meta-analysis by Gates et al in 2015 did not show that an advantage to the use of mechanical chest compression devices for ROSC, survival to discharge from hospital or 30 days and survival with good neurological outcome.^[29]^ A study by Li et al in 2016 showed that there was no significant difference in admission survival rate, discharge survival rate and CPC score between manual compression and mechanical compression, and that manual compression was superior to mechanical compression in ROSC.^[30]^ And in this meta-analysis, we found that LUCAS has no advantage over manual CPR. The results is consistent with the above research in ROSC, survival to hospital admission, survival to hospital discharge, and survival to 30 days. Compared with the above 2 meta-analysis, this study included more studies and included more subjects; in addition, this study only discussed the results with LUCAS, without other mechanical CPR, which reduce the bias from other machines. Therefore, this meta-analysis is more reliable.

We had the following assumption for this situation: first, LUCAS lacks the simplicity and timeliness of manual chest compression. As mentioned above, the delay of chest compression and interruption of chest compression will lead to a decrease in CPR quality. When a CA patient is encountered, manual chest compression can begin immediately, while LUCAS will delay the time of CPR due to the assembly by professional. Second, the depth of chest compression with the LUCAS is 4 to 5 cm, which is less than 5 cm as defined by the guideline. It will lead to invalid CPR. Giraud et al performed a study of the effectiveness of LUCAS in 2015 which showed that it was ineffective with the LUCAS by transesophageal echocardiography.^[31]^ Therefore, the application of LUCAS is limited due to these shortcomings. Some studies even showed that manual CPR is better than LUCAS.^[30,32]^

### 4.2 Strengths

Compared with the previous study, this paper, a meta-analysis of 6 studies, including more subjects, is a large sample survey. In addition, the studied indicators in this paper include not only the success rate after CPR, but also the short-term outcome indicators such as ROSC, survival to hospital admission, survival to hospital discharge and survival to 30 days of patients. It ensure this meta-analysis higher credibility. And the subjects in this meta-analysis come from various country, therefore, the results of this study have a wide range of applicability. Hence, this study may help to choose the ways of CPR. Manual CPR is still good for emergencies; in the case of prolonged compression, LUCAS may be a better alternation than manual PCR.

### 4.3 Limitations

There were also some limitations in this meta-analysis. This article contains a prospective observational study and a descriptive, nonrandomized controlled trial, which may cause some bias due to the heterogeneity of research; second, all included studies failed to do blind method due to the need to rescue patients. Third, the included studies lacked data which can evaluate quality indicators in the process of CPR (such as end-tidal carbon dioxide, degree of blood oxygen saturation, etc), cerebral performance category, and neurological recovery, and so on. Therefore, these data were not analyzed. But a study by Rubertsson 2014 in suggested that there was no significant difference between manual CPR and Mechanical chest compression with LUCAS in 4-hour survival.^[33]^ Finally, all subjects were adults in this study, and age stratification was not analyzed, as a result, the results of this study only apply to adults.

### 4.4 Future directions

Manual CPR and LUCAS have their own advantage and defect respectively, but the study found no significant difference in clinical outcomes of patients. It showed that the LUCAS is a practical tool, with a similar clinical outcome to manual CPR. Therefore, more research is needed to confirm this in the future. Moreover, it was not studied that whether the way combined manual CPR with LUCAS can improve the clinical outcome of CA patients. Future research can analysis it and explore the situation of using LUCAS.

## 5 Conclusion

In this systematic review, combined with a meta-analysis of related data, it is found that there is no significant difference between manual chest compression and LUCAS in improving clinical outcomes in patients with out-of-hospital CA. More large-scale studies are needed in the future.

## Author contributions

**Conceptualization:** Mao Liu.

**Investigation:** Zhuang Shuai, Kai Tang, Jiankang Zheng.

**Methodology:** Mao Liu, Zhuang Shuai, Jiao Ai, Hui Liu.

**Project administration:** Mao Liu, Zhan Lv.

**Resources:** Zhuang Shuai, Kai Tang, Jiankang Zheng.

**Software:** Mao Liu, Zhuang Shuai, Jiao Ai, Hui Liu, Junqi Gou.

**Supervision:** Mao Liu, Hui Liu, Zhan Lv.

**Validation:** Mao Liu, Zhan Lv.

**Writing – original draft:** Mao Liu, Zhuang Shuai.

Mao Liu orcid: 0000-0001-5622-5178.

## Supplementary Material

Supplemental Digital Content
