# Supplementary material for: Mechanical chest compression with LUCAS device does not improve clinical outcome in out-of-hospital cardiac arrest patients: A systematic review and meta-analysis
Source: Medicine (Baltimore). 2019 Nov 1;98(44):e17550. doi: 10.1097/MD.0000000000017550 (PMC6946388; doi:10.1097/MD.0000000000017550)
Supplement: Supplemental Digital Content [file medi-98-e17550-s001.docx]

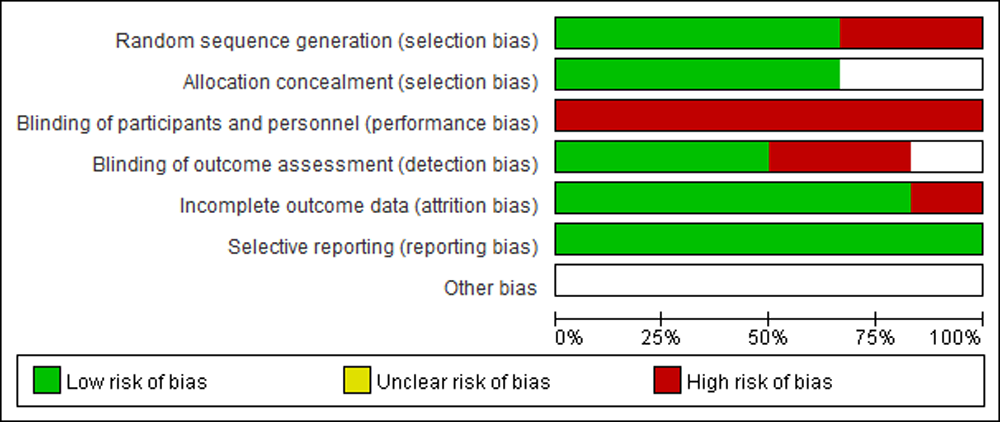


**Suppl.1** Risk of bias graph. Each risk of bias item presented as percentages across all included studies.

**Suppl.2** Results of univariable meta-regression analysis.

| **LUCAS vs Manual** | **Studies** | **SE** | **P value** | **95% CI** |
| --- | --- | --- | --- | --- |
| **Sample size, MD** | 6 | <0.001 | 0.612 | -0.0006, 0.0004 |
| **Research type, RR** | 6 | 0.773 | 0.756 | -2.4045, 1.8894 |
| **Publication year, MD** | 6 | 0.108 | 0.774 | -0.2669, 0.3332 |
| **Race, MD** | 6 | 0.620 | 0.536 | -2.1402, 1.3022 |

Research types included RCT and non-RCT. Races included Caucasian and Mongolian in this meta-analysis. MD, mean difference; RR, relative risk; SE, standard error; CI, confidence interval; RCT, randomized controlled trial.
